# Infertility Among Male Patients With Tramadol abuse

**DOI:** 10.1192/j.eurpsy.2023.213

**Published:** 2023-07-19

**Authors:** A. W. Abouhendy, M. M. Bassiony, U. M. Youssef, H. M. Elgohary

**Affiliations:** 1 Addiction Unit, Psychological Medicine Hospital, Cairo; 2 Addiction Unit, Zagazig university, Zagazig, Egypt

## Abstract

**Introduction:**

Tramadol abuse has become a crisis in Egypt and many other Middle Eastern countries. Tramadol abuse is associated with sexual dysfunction and male infertility, according to recent animal and human studies.

**Objectives:**

The objective of this study was to compare tramadol abuse patients and healthy controls regarding free testosterone and prolac-tin levels and semen analysis.

**Methods:**

Sixty patients with opiate use disorders attributed to tramadol (OUD-T) based on Diagnostic and Statistical Manual of Mental Disorders (Fifth Edition) diagnostic criteria and 30 healthy controls were included in the study. Sociodemographic and clinical data and urine, blood, and semen samples were collected from patients and controls for assessment.

**Results:**

Compared with controls, OUD-T patients had higher prolactin and lower free testosterone levels. Patients with OUD-T were more likely to have lower sperm count and higher abnormal motility and forms of sperms compared with controls.

**Conclusions:**

Patients with OUD-T were found to be more likely to have lower free testosterone levels and lower sperm counts and vitality, and higher prolactin levels and sperm abnormal forms compared with controls.

**Disclosure of Interest:**

None Declared

